# Identification of Dynamic Characteristics of High-Rise Buildings Considering the Influence of Modal Direction

**DOI:** 10.3390/s26144365

**Published:** 2026-07-09

**Authors:** Yinghou He, Pakwai Chan, Yujie Liu, Biao Hu, Shuai Teng, Qiusheng Li

**Affiliations:** 1 Research Center for Wind Engineering and Engineering Vibration, Guangzhou University, Guangzhou 510006, China; heyinghou@gzhu.edu.cn (Y.H.); yjliu@gzhu.edu.cn (Y.L.); 2 Hong Kong Observatory, Kowloon, Hong Kong 999077, China; pwchan@hko.gov.hk; 3 Guangdong Provincial Key Laboratory of Durability for Marine Civil Engineering, Shenzhen University, Shenzhen 518060, China; 4 School of Intelligent Construction and Civil Engineering, Zhongyuan University of Technology, Zhengzhou 450007, China; tengs@zut.edu.cn; 5 Department of Architecture and Civil Engineering, City University of Hong Kong, Hong Kong 999077, China; bcqsli@cityu.edu.hk

**Keywords:** high-rise building, super typhoon, field measurement, wind-induced vibration response, modal direction, modal identification

## Abstract

The structural dynamic characteristics of super high-rise buildings are key to understanding how they respond to wind-induced vibration. Currently, one widely adopted method involves using vibration sensors to capture structural vibration responses through on-site measurements, followed by identifying structural dynamic characteristics using output-only methods. However, when measuring and analyzing the dynamic characteristics of super high-rise buildings, the modal directions associated with the vibration modes of the structure are often ignored, which can lead to identification errors. This is particularly true for super high-rise buildings with irregular cross-sections, for which research into the impact of actual structural vibration modes is notably lacking. Therefore, this study uses a normal mode decomposition method to examine the determination of structural vibration mode directions in detail. This method identifies the angular deviation between the viewing and normal coordinates by analysing the spectral energy distribution. It then decomposes the measured signal in the viewing coordinate system based on this deflection angle. This achieves decoupling of modes that are coupled in both directions. Specifically, the study analyses the phenomenon of modal aliasing in structural modal parameters from both time-domain and frequency-domain perspectives based on the measured acceleration response signals of a super high-rise building with a non-circular cross-section during Super Typhoon Saola, and employs the structural modal orthogonal decomposition method to determine the modal directions. The fundamental sway modes of the structure exhibit aliasing between the two adjacent modes at 0.1748 Hz and 0.1825 Hz due to a 46° angle of deviation between the viewing and normal coordinates. Based on the clarification of modal directions, the study further refines the identification of structural modal parameters. Following decoupling, the dispersion of the damping ratio and frequency identification results in the normal coordinate system decreased significantly (concentrating at 1.0–2.0% and 0.175–0.185 Hz, respectively). The damping ratio increased by 1.0% with increasing amplitude, while the frequency decreased by 0.005 Hz with increasing amplitude. The research findings help to improve the accuracy with which the dynamic characteristics of super high-rise buildings can be identified, thereby enabling the structure’s wind-induced response to be assessed more rationally.

## 1. Introduction

According to the latest statistics from the Council on Tall Buildings and Urban Habitat (CTBUH), China currently ranks first in the world in the number of completed high-rise buildings. However, high-rise buildings are extremely sensitive to wind loads due to their high flexibility and low natural vibration frequencies. China’s coastal regions, which have a large number of high-rise buildings, are particularly vulnerable to strong winds and typhoons. High-rise buildings typically have a long service life, with significant super high-rise buildings designed to last up to 100 years. During this period, structural damage and resistance degradation are inevitable due to the combined effects of environmental erosion, material ageing, long-term load effects and other factors [1]. Structural modal parameters are of great significance as the prerequisite information for the wind and seismic design of high-rise buildings. The performance degradation or damage of high-rise buildings is a long-term cumulative process that spreads from local components to the entire structure [2,3]. During this process, physical parameters such as structural stiffness and mass change, resulting in time-varying modal parameter characteristics [4,5]. Therefore, accurately analysing the dynamic characteristics of the structure and obtaining key modal parameter information is essential.

Structural modal identification technology can accurately determine the modal parameters of high-rise buildings. This process involves solving for modal parameters such as structural frequency, damping ratio, and mode shape, based on input data (external excitation) and output data (structural response). According to whether known input information is required, structural modal identification can be divided into two categories: Experimental Modal Analysis (EMA) and Operational Modal Analysis (OMA). OMA can estimate the dynamic characteristics of structures by relying solely on structural vibration responses. This method is advantageous as it is convenient and rapid and does not impact the normal use of structures. It has therefore been widely used in the modal identification of engineering structures [6].

Engineering structures often undergo changes in dynamic characteristics during long-term operation under continuous external load excitation [7]. Scholars have conducted extensive research into the variation in structural modal parameters based on time-varying and time-invariant structure assumptions, respectively. In their study of the dynamic characteristics of time-invariant structures, Jeary [8] applied the Random Decrement Technique (RDT) to identify the modal parameters of high-rise buildings, using this method to describe the frequency and damping ratio amplitude dependence of such buildings. Gu et al. [9] combined the Hilbert–Huang transform (HHT) with the RDT method using field-measured data from the Jin Mao Tower in Shanghai. They identified the first ten modal parameters of the structure and found that the standard deviation of the structural damping ratio increased as the modal order increased. Shi et al. [10] compared the identification results for the first 11 modal frequencies and damping ratios of the Shanghai World Financial Center obtained using the Peak Picking (PP) method and the HHT, based on small-amplitude vibration records of the building under environmental excitation. They found that the two methods produced highly consistent results. Li et al. [11] used the Natural Excitation Technique (NExT), combined with the Eigensystem Realization Algorithm (ERA), to identify the structural modal parameters of a high-rise office building. They then compared and analysed the amplitude dependence of the first three modes in detail and proposed an empirical formula relating the structural top drift ratio to the structural frequency. He et al. [12] used the Bayesian Spectral Density Approach (BSDA) and RDT to identify the modal parameters of a high-rise building under typhoon excitation, and compared the advantages and disadvantages of the two methods. He et al. [13] incorporated the energy grouping technique into Covariance-Driven Stochastic Subspace Identification (SSI-COV) to enhance the accuracy of modal parameter identification in high-rise buildings subjected to typhoon action. Zhou et al. [14] analysed the structural dynamic characteristics of the Ping-An Finance Centre in Shenzhen in its two operating states (with and without damper) using the RDT and SSI-COV methods and evaluated the damper’s performance accordingly. In their research into the dynamic characteristics of time-varying structures, Chen et al. [15] verified the accuracy and anti-noise performance of a time-varying system modal parameter identification method based on multi-scale chirplet sparse signal decomposition using a numerical example. Guo and Kareem [16] proposed a non-stationary system identification method based on wavelet transform to analyse the time-varying characteristics of structural frequency and damping. Liu et al. [17] successfully identified the instantaneous frequency of time-varying structures by combining wavelet transform and variational mode decomposition. Ni et al. [18] enhanced the variational mode decomposition technique and demonstrated its applicability in identifying time-varying structural modal frequencies through numerical simulations and experiments. Yao et al. [19] proposed an autoregressive, spectrum-guided variational mode decomposition method, demonstrating its effectiveness in identifying time-varying structural modal parameters under non-stationary excitation through numerical simulation.

Modal aliasing, i.e., dense frequency distribution, is a widespread phenomenon in the time-varying and time-invariant structural modal analysis of high-rise buildings, and cannot be ignored [20,21]. However, research into accurately identifying and understanding the mechanisms of modal aliasing is insufficient. Ignoring or misjudging modal aliasing often has a significant impact on the accuracy of modal identification results [22,23,24]. Due to the complex structural systems of high-rise buildings, the geometric axis of the structure may not align with the modal vibration direction. This inconsistency leads to coupling between modes, causing modal aliasing and having a significant impact on modal identification [25,26].

To address this problem, researchers have adopted two-degree-of-freedom RDT and modal decomposition methods to decouple aliased modes (see references [27,28,29]). Zhou et al. [30] proposed a modal decomposition method based on normal coordinates for decoupling closely coupled modes. Subsequently, Li et al. [26] used the modal decoupling method combined with RDT to identify the dynamic characteristics of four super high-rise buildings. However, research into this key issue remains relatively limited, particularly with regard to high-rise buildings featuring special cross-sections. These buildings have more diverse cross-sectional forms than conventional rectangular or square ones, and the correlation mechanism between the geometric principal axis and the vibration modal direction is more complex. The relevant influence mechanism needs to be clarified urgently.

In addition, the modal parameters of high-rise buildings under large amplitudes induced by strong winds often show unique nonlinear evolution characteristics, compared with the structural vibration behaviour under medium and small amplitudes. Under these extreme working conditions, further study is necessary to clarify the evolution law of structural modal parameters under large-amplitude excitation in the context of modal aliasing. Therefore, this study takes the Zhuhai Center Tower (ZHCT) as its research subject. This building has a unique geometric shape, featuring an arc-shaped cross-section and an overall spiral design, and is a prime example of a super-high-rise building with a special cross-section. Based on field-measured wind-induced response data obtained from the building during Super Typhoon Saola, this study systematically discusses the generation mechanism of modal aliasing and focuses on analysing the influence of dense frequency distribution on structural dynamic characteristics. On this basis, it finely identifies the structural modal parameters.

This study aims to reveal the modal aliasing and beat frequency effects caused by misalignment between modal directions and the observation coordinate system in super high-rise buildings, particularly those with irregular cross-sections. It also applies and expands upon an accurate decoupling method based on regular modal decomposition. Unlike existing research, which either focuses on buildings with regular cross-sections or ignores directional deviations entirely, this paper uses the ZHCT, which has an arched, spiral cross-section, as its subject. Based on field measurements taken during Super Typhoon Saola, it systematically elucidates how two adjacent modes (0.1748 Hz and 0.1825 Hz) generate spectral bimodality and time-domain beats (with a period of approximately 260 s) when there is a 46° deviation angle between the observation coordinate system and the principal axes of the structure’s regular modes. The modal direction identification and coordinate rotation transformation techniques effectively separate closely coupled modes, significantly reducing the discretization of the decoupled damping ratio and frequency identification. This clearly reveals amplitude-dependent evolution patterns, showing that the damping ratio increases whilst the frequency decreases with increasing amplitude. This paper innovatively incorporates the often-overlooked factor of ‘modal orientation’ into the framework for identifying dynamic responses, providing a more precise theoretical foundation and technical approach for evaluating the aeroelastic response of super high-rise buildings with complex cross-sections.

## 2. Observation Platform and External Excitation Conditions

### 2.1. Structural Health Monitoring System of the Monitored Building

The ZHCT, located in the cross-border central business district of Zhuhai City in Guangdong Province, is a 330-metre-high skyscraper with a streamlined glass curtain wall facade and an overall slender, spiral shape that exhibits significant geometric irregularity. As a landmark building in Zhuhai, the ZHCT is not only the core carrier of regional business and commercial activities, but also an ideal subject for studying the wind-induced effects of super high-rise buildings with irregular cross-sections in structural wind engineering, thanks to its unique geometric shape and height. The building’s structural dynamics and wind vibration control under strong winds have attracted significant interest.

**Figure 1:**
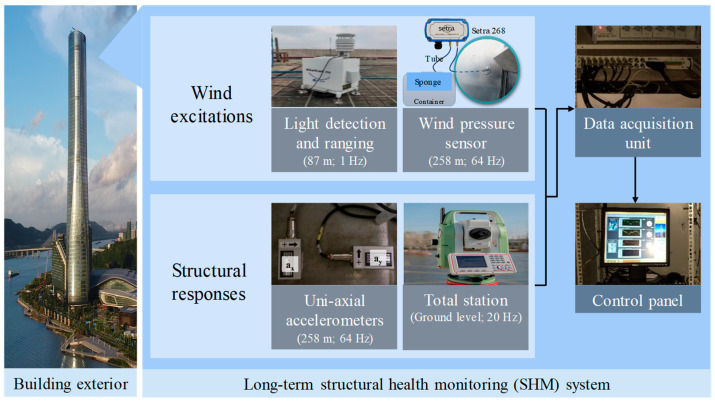
Structural health monitoring system for the ZHCT.

In order to systematically study the wind-induced vibration characteristics of the ZHCT in strong winds, the research team set up a long-term structural health monitoring system (see Figure 1). This system comprises two main modules: a wind load excitation monitoring module and a structural response monitoring module. The former obtains aerodynamic load information acting on the building surface, while the latter collects information on the structural dynamic response caused by it. For wind load monitoring, a LiDAR sensor was installed on top of an adjacent building to collect wind field information around the ZHCT, including key parameters such as average wind speed, wind direction and air pressure. To further obtain the spatiotemporal evolution characteristics of wind pressure distribution on the building surface, multiple Setra 268 capacitive wind pressure sensors were arranged at a height of 258 m (the 56th floor), effectively capturing the transient process of fluctuating wind pressure. For structural response monitoring, two pairs of velocimeters and accelerometers were installed at 258 m to continuously record the building’s response to wind-induced vibration. Meanwhile, to obtain the displacement response of the building’s top, a total station is set up at a ground reference point to track the building top’s three-dimensional spatial displacement in real time.

**Figure 2:**
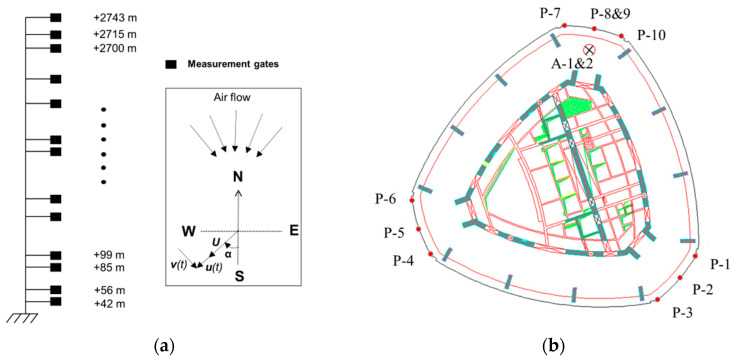
Layout of measurement points on LiDAR (**a**) and ZHCT (**b**). (P-1 to P-10 denote pressure transducers and A-1 & 2 denote accelerometer and velocimeter, respectively).

The vertical distribution of measurement points for the LiDAR and the ZHCT is shown in Figure 2 and summarized in Table 1. The LiDAR measurement range was configured from 42 m to 2743 m with a height resolution of 15 m. On the 56th floor of the ZHCT, sensors were deployed to record wind effect information, enabling an investigation into the characteristics of the wind field, the pressures on the building cladding, and the responses of the structure to vibration.

All data collected by the sensors are transmitted to the data acquisition unit via wired or wireless means. After preliminary processing, the data is uniformly uploaded to the control centre’s data management and analysis platform. This platform offers functions such as data storage, synchronous display, abnormal alarms and offline analysis. It enables long-term, continuous and synchronous monitoring of building wind loads and structural responses. This system enables researchers to obtain high-quality measured data covering multiple typhoon events, providing a solid basis for in-depth discussion of the wind vibration characteristics of super high-rise buildings in strong wind environments.

### 2.2. Super Typhoon Saola

**Figure 3:**
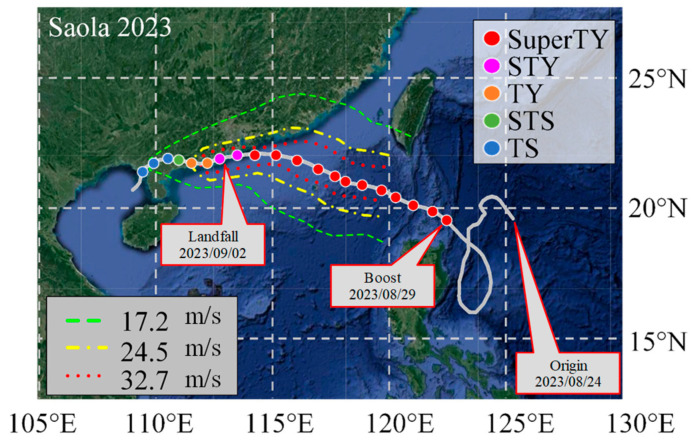
Typhoon Saola’s track and characteristics.

Super Typhoon Saola, the ninth typhoon of 2023, was a strong storm that affected the coastal areas of southern China. Figure 3 shows its complete life history and movement track. The typhoon formed over the north-west Pacific Ocean on 24 August, initially moving north-westwards as a tropical storm. From 29 August, Saola entered a rapid intensification period, developing into a strong typhoon and then a super typhoon within a short period of time. The continuous red marks on the path map reflect the process of its intensification. During its movement, the typhoon’s path underwent a cyclonic phase and an adjustment phase, after which it resumed stable northwestward movement and made landfall along the coast of Guangdong on 2 September. On the eve of landfall, Saola maintained high intensity, making it one of the strongest typhoons to affect South China that year. The isolines of different colours in the figure clearly depict the spatial distribution characteristics of Saola’s wind field, showing its large-scale, strong wind circulation structure. The typhoon caused significant wind damage to coastal areas along its path and in South China, particularly in the form of sustained high-speed winds and changes in wind direction.

### 2.3. Incoming Wind Speed and Direction

**Figure 4:**
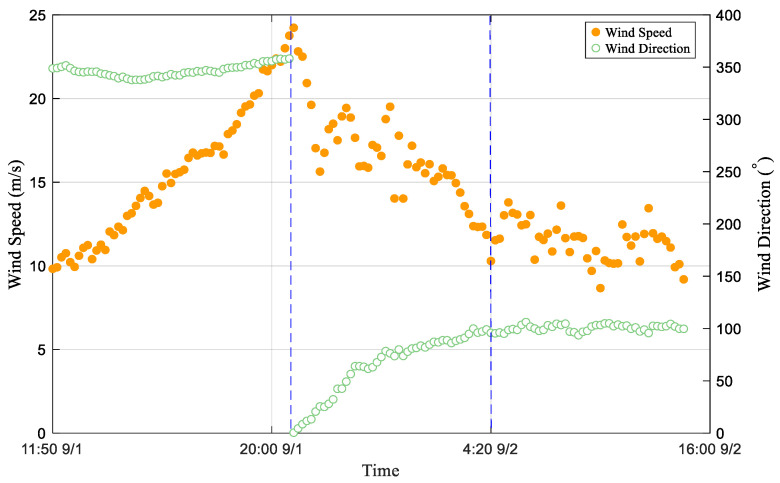
Incoming wind speed and direction records during Typhoon Saola (the dotted lines show where the wind direction changes abruptly).

**Figure 5:**
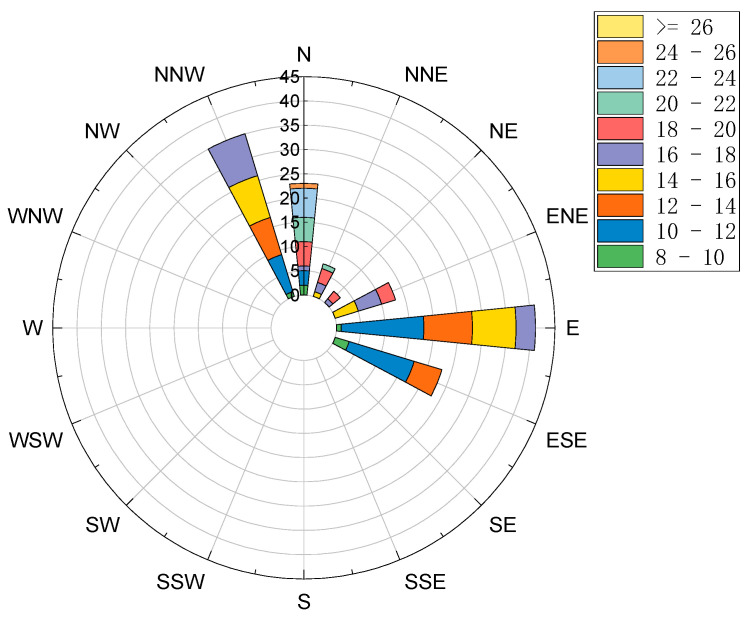
Wind rose diagram of prevailing winds during Typhoon Saola.

Figure 4 shows the complete time-history evolution of the measured 10 min average wind speed and wind direction at a height of 285 m (the 56th floor) of the ZHCT during the influence of Typhoon Saola, reflecting the evolution characteristics of the wind field before and after the typhoon passage. Additionally, Figure 5 presents a wind rose diagram to illustrate the changes in wind direction more clearly, showing the development of the prevailing winds during the typhoon. From 11:50 on 1 September, as the typhoon centre gradually approached, the measured wind speed showed a significant upward trend, steadily increasing from an initial speed of around 10 m/s. After 20:00 on 1 September, the wind speed rapidly peaked, with the maximum average wind speed reaching 25 m/s. During the period when the typhoon centre was directly overhead, the wind speed remained in the range of 15–20 m/s, exhibiting high intensity and high variability. Afterwards, as the typhoon moved away, the wind speed gradually decreased from early morning on 2 September, dropping to around 10 m/s by the afternoon. The overall evolution of the wind speed is highly synchronised with the movement path and intensity changes in the typhoon. The change in wind direction shows the typical characteristics of a typhoon wind field. Initially, under the influence of the typhoon, the wind direction was stable at approximately 350° (northerly wind). However, at the critical moment when the typhoon centre approached, there was a sharp clockwise deflection in the wind direction, turning rapidly from 350° to approximately 100° (easterly wind). This deflection process was accompanied by violent fluctuations in wind speed, clearly depicting the asymmetric wind field structure of the typhoon vortex and the step-like changes in wind direction during its passage.

## 3. Analysis and Discussion

### 3.1. Structural Wind-Induced Dynamic Response

**Figure 6:**
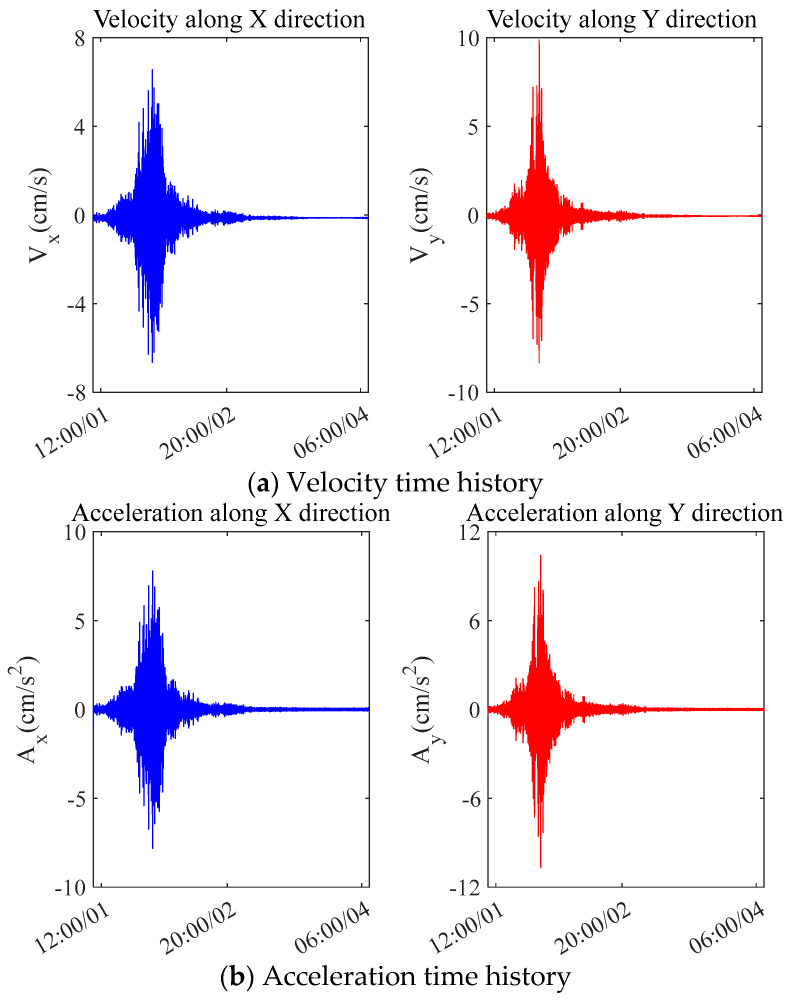
Measured structural vibration responses.

Figure 6 shows the time-history curves of the wind-induced dynamic response (velocity and acceleration) at the 56th floor (258 m) of the ZHCT during Typhoon Saola. The velocity response time history (Figure 6a) exhibits significant non-stationary, time-varying characteristics in both the X-direction and Y-direction (denoted as east–west and north–south directions, respectively). The maximum velocity amplitude in the X-direction is approximately 7 cm/s, whereas the peak value in the Y-direction is around 10 cm/s. This significant difference reveals the building’s anisotropic dynamic behaviour under the typhoon flow field. The peak velocity response values are concentrated between the night of 1 September and the early morning of 2 September, which is highly consistent with the time at which the centre of Typhoon Saola approached the building. This indicates a direct temporal correlation between the structural velocity response and the evolution of typhoon wind load intensity. After 20:00 on 2 September, as the typhoon gradually moved away, the velocity response rapidly attenuated and stabilised. The acceleration time-history curve (Figure 6b), being the time derivative of the velocity response, exhibits a highly synchronised time-varying trend with the velocity response. The peak acceleration in the X-direction is approximately 8 cm/s^2^, while that in the Y-direction is around 12 cm/s^2^, consistent with the anisotropic characteristics of the velocity response. The more significant acceleration response in the Y-direction indicates that wind-induced vibration in this direction generates a higher inertial force, which significantly impacts the evaluation of structural comfort and safety.

It is worth noting that the response in the Y-direction is significantly stronger than in the X-direction. This difference may be due to the relationship between the building’s asymmetric cross-section and the typhoon’s dominant wind direction. It also suggests that the dynamic amplification effect in specific directions should be considered in wind-resistant design.

### 3.2. PSD Analysis of Acceleration Time History

**Figure 7:**
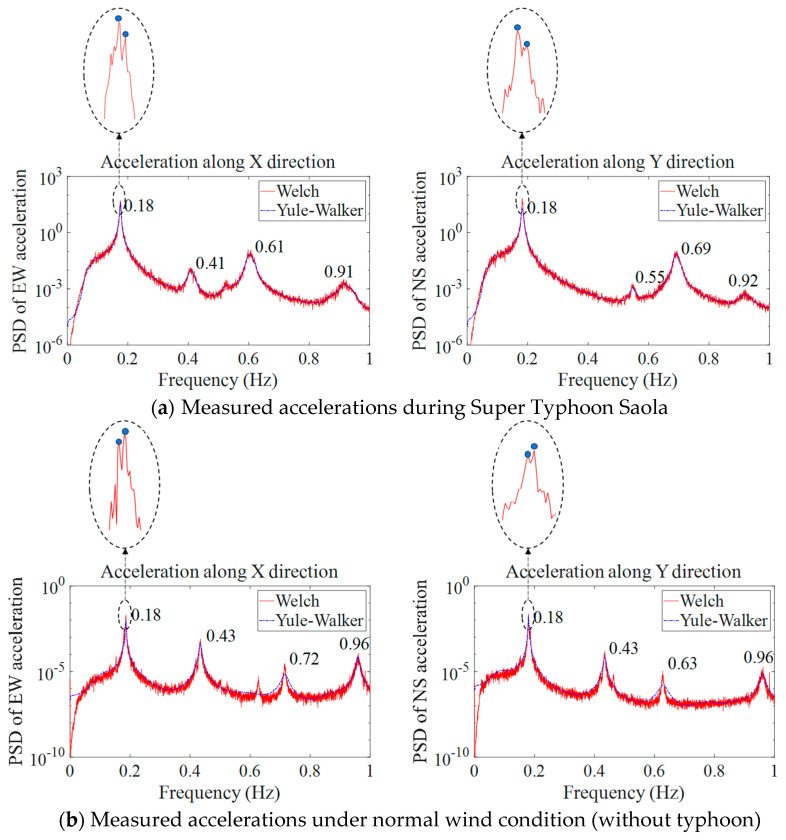
Power Spectral Density (PSD) analysis of acceleration time history.

Figure 7 presents the spectral analysis results of the structural vibration response during a typhoon and under normal wind conditions, respectively. The data segments for these two conditions correspond to the structural vibration response under the influence of Super Typhoon Saola (see Figure 6) and to the response recorded under ambient excitation (on 30 May 2026 from 12:00 to 22:00), respectively. Figure 7a shows the structural response spectrum during the typhoon, while Figure 7b shows the spectrum under normal wind conditions.

Power spectral density (PSD) analysis of the ZHCT acceleration response in both the X and Y directions can reveal its multimodal dynamic characteristics. The PSD results under typhoon and normal wind conditions revealed that cross-validation using the Welch and Yule–Walker methods showed highly consistent overall trends in the spectral curves, thereby verifying the robustness and reliability of the analytical results.

As Figure 7a,b show, near the structure’s first-order sway mode frequency (approximately 0.18 Hz), the spectra for both wind scenarios exhibit significant local bimodality. This bimodal characteristic is indicative of the aliasing effect of the first-order sway mode, suggesting that under strong typhoon excitation or in normal wind conditions, the structure’s first-order modal response is often due to the contribution of two coupled modes with similar frequencies rather than a single mode.

In addition to the first-order mode, the spectral curves clearly show the contributions of several higher-order modes. Based on the PSD amplitude levels, it can be seen that the structural vibration energy is significantly greater during a typhoon than in normal wind conditions. Furthermore, during the typhoon period, the higher-order modes are 0.41, 0.61 and 0.91 Hz in the east–west (EW) direction and 0.55, 0.69 and 0.92 Hz in the north–south (NS) direction. Under normal wind conditions, the higher-order modes are uniformly 0.43, 0.63/0.72 and 0.96 Hz in the EW and NS directions. The higher-order modal frequencies of the structural response to typhoon excitation are generally lower, reflecting the softening effect of large deflections induced by strong winds on structural stiffness.

### 3.3. Causes of Modal Aliasing

An important cause of modal aliasing in high-rise buildings is the geometric misalignment between the structural normal coordinate system and the observation coordinate system, as well as the structure’s inherent dynamic anisotropy [30,31]. In super high-rise buildings with square cross-sections, the two orthogonal fundamental modal frequencies are close together, easily inducing beat effects.

**Figure 8:**
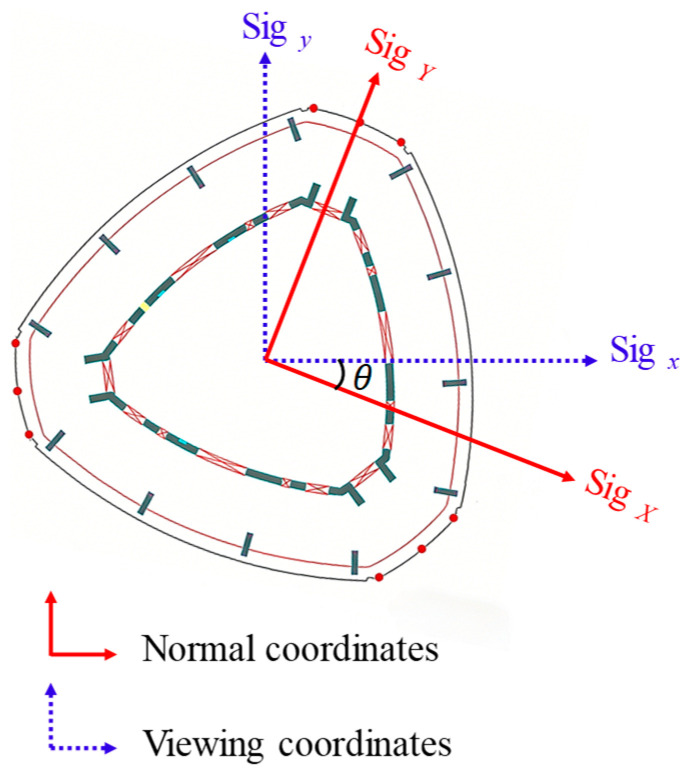
Schematic diagram of structural vibration mode direction (x and X are associated with the east–west direction, while y and Y are related to the north–south direction).

As illustrated in Figure 8, buildings are often asymmetrically laid out in engineering practice, resulting in an included angle θ between the viewing coordinate system (Sigx/Sigy), which is based on the natural geometric axis, and the real orthogonal fundamental modal direction of the structure (normal coordinate, SigX/SigY). In the normal coordinate system, the vibration of each mode is an independent single-mode response [32]. However, when measured through the observation coordinate system, the normal vibration components are projected onto the observation axes, resulting in the energy of two orthogonal modes appearing in the response signal of an observation direction simultaneously. This mixes the modal contributions of two similar frequencies in the acceleration time history of a single observation direction, ultimately showing frequency aliasing in the spectrum analysis [33]. This mechanism interferes with the accurate identification of modal parameters and has an adverse effect on the evaluation of structural wind-induced vibration response.

### 3.4. Determining the Occurrence of Modal Aliasing in the Frequency-Domain

To clarify the specific components of the coupled modes in the fundamental frequency mode, this study designed a zero-phase digital filter to filter the acceleration signal and extract the contribution of the fundamental sway mode. According to the multi-modal analysis results mentioned above (Figure 7), the passband of the filter was set to 0.14–0.22 Hz, which completely covers the expected frequency range of the first-order sway mode of the structure.

**Figure 9:**
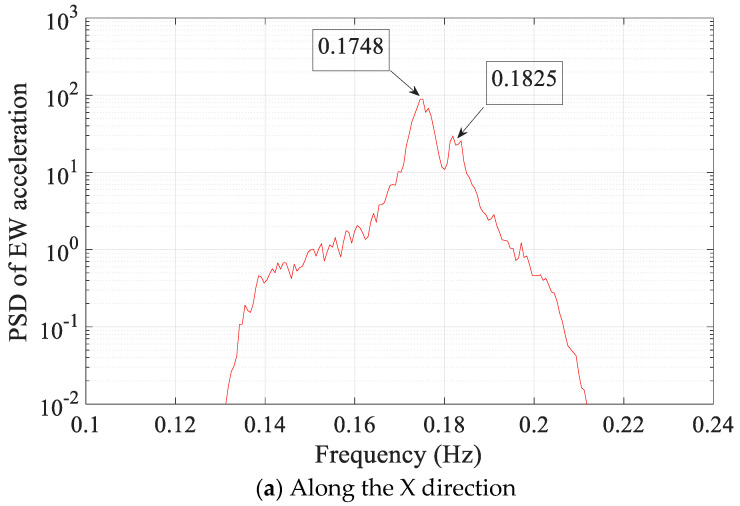

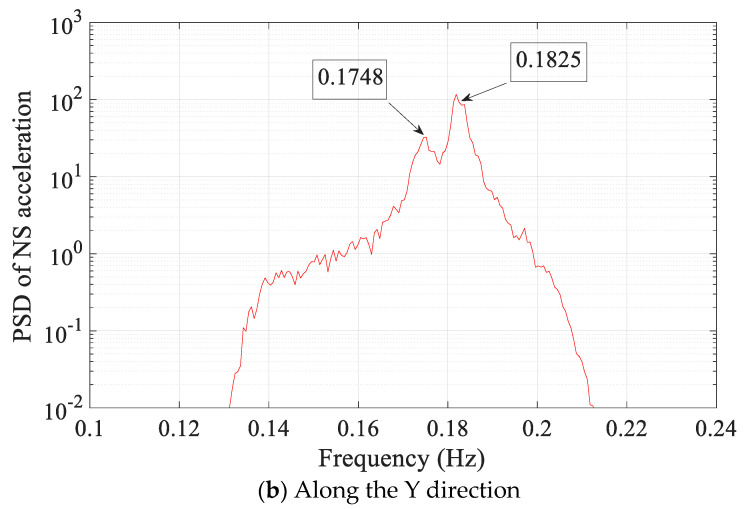
Observations of beating in the frequency-domain.

PSD analysis was carried out again based on the fundamental mode time history obtained after filtering. Figure 9 shows the fundamental mode acceleration spectra in the east–west and north–south observation directions.

The PSD curve in the X direction shows two significant energy peaks in the target frequency band, with central frequencies of 0.1748 Hz and 0.1825 Hz respectively. The 0.1748 Hz peak is the main energy peak and dominates the fundamental frequency response in this direction. Similarly, the PSD curve in the Y direction also identifies these two frequency components, but the energy distribution shows significant differences. The 0.1825 Hz frequency becomes the main peak in this direction while the 0.1748 Hz frequency drops to a secondary peak. The difference between the two frequencies is only 0.0077 Hz, which is very close to the typical frequency spacing between the two orthogonal fundamental sway modes of super high-rise buildings.

These double-peak features confirm the aforementioned modal aliasing mechanism. The first two-order sway modes, vibrating independently in the normal direction, are simultaneously projected onto the corresponding directions in the observation coordinate system. This results in the response signals of both directions mixing the energy components of the two modes. The difference in the main energy peaks in the two directions indicates that the projection contributions of the normal modes in different observation directions differ; that is to say, the X direction has a higher proportion of projection of the 0.1825 Hz mode, while the Y direction is dominated by the 0.1748 Hz mode.

### 3.5. Determining the Occurrence of Modal Aliasing in the Time-Domain

In terms of time-domain analysis, this study used the Random Decrement Technique (RDT) to identify the beat phenomenon in the acceleration of the fundamental frequency mode. According to previous frequency-domain analysis results, the first-order sway mode of the building is coupled with two modes that have frequencies very close to each other (0.1748 Hz and 0.1825 Hz), so its time-domain response is expected to exhibit typical beat oscillation characteristics.

In order to fully capture the evolution process of the beat effect, the time parameter τ (i.e., the intercept length of the random decrement signal) in RDT is set to between 60 and 80 times the period of the fundamental mode. As the period of the fundamental mode can be taken as the average of the two coupled modes (about 5.65 s), we have that 340 ≤ τ ≤ 450, which is long enough to cover multiple complete beat periods.

Additionally, according to the study by Çelebi et al. [22], when two adjacent modes are coupled, the beat period of their free decay signal can be calculated using the following formula:

(1) $$T_{b}=\frac{2}{f_{b}}=\frac{2}{\left|f_{1}-f_{2}\right|}=\frac{2T_{1}T_{2}}{\left|T_{1}-T_{2}\right|}$$

where T_b_ and f_b_ are the beat period and frequency, respectively, and f_b_ is the absolute value of the difference between the two coupled mode frequencies (f_1_ and f_2_). According to Equation (1), the beat period of the fundamental sway mode of the ZHCT can be obtained as:

(2) $$T_{b}=\frac{2}{\left|f_{1}-f_{2}\right|}=\frac{2}{\left|0.1825-0.1748\right|}=260(s)$$

**Figure 10:**
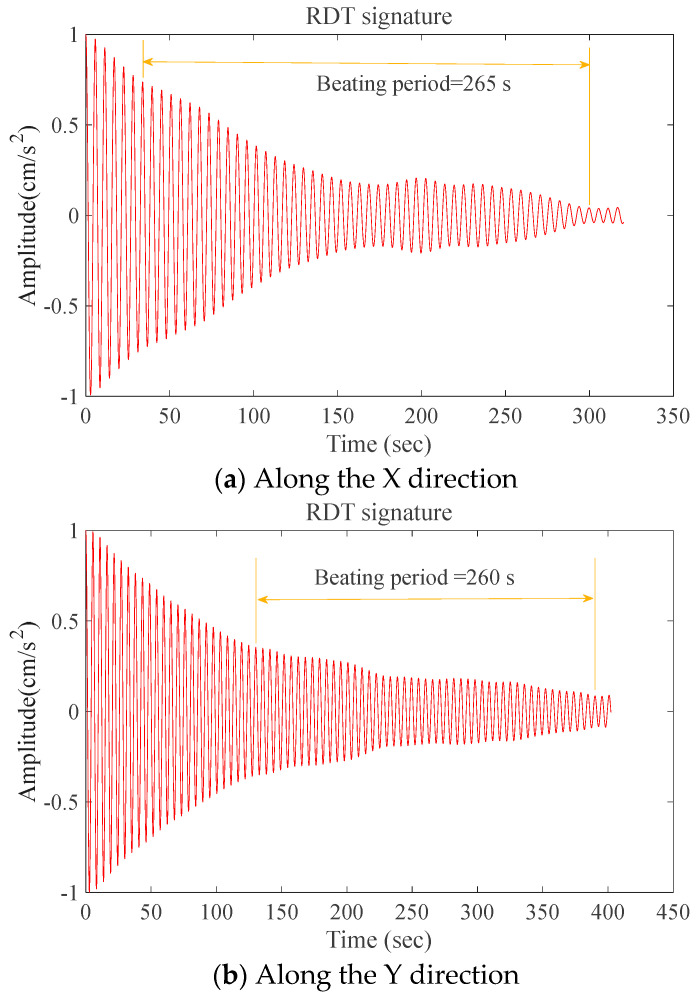
Observations of beating in the time-domain.

The results of the analysis are shown in Figure 10. The free decay signals in both directions exhibit the characteristic shape of beat oscillation. Specifically, the X-direction signal shows beat evolution characteristics, with the amplitude first decaying and then slowly rising over a period of 265 s. The Y-direction free decay signal shows that the amplitude rapidly decays over time in the initial stage. It then enters a periodically modulated oscillation stage, completing a full ‘decay-enhancement’ cycle over a period of about 260 s. The calculation beating results are listed in Table 2. The RDT analysis results are highly consistent with the modal coupling theoretical prediction, with a maximum deviation of no more than 5%.

In summary, the results of the RDT time-domain analysis and the double-peak features observed in the previous PSD frequency-domain analysis provide mutual verification. Together, they clarify the measured period of the beat phenomenon and reveal differences in the degree of modal coupling in different observation directions.

### 3.6. Normal Mode Decomposition Method

The core objective of this method is to decouple the coupled measured responses and restore the uncoupled, orthogonal modal characteristics of the structure within the normal coordinate system. Based on the principle of coordinate rotation transformation and the orientation shown in Figure 6, the method takes the measured acceleration responses in the observation coordinate system (Sigx, Sigy) as input and uses the rotation transformation defined by Equation (3a,b) [34] to construct the directional dynamic responses SigX and SigY, which vary with the rotation angle θ.

(3a) SigX=Sigx·cosθ+Sigy·sinθ

(3b) SigY=−Sigx·sinθ+Sigy·cosθ

where SigX and SigY are the acceleration responses in the modal direction, and Sigx and Sigy are measured responses along the viewing coordinates.

In the normal coordinate system of the structure, the first two sway modes are orthogonal to each other, meaning that the response in each direction contains only a single fundamental frequency. The spectrum energy is also highly concentrated. In contrast, the response under the observation coordinate system is the superposition of projections of the two normal modes. This results in two frequencies being present at the same time, which shows modal aliasing and the beat effect. Therefore, decomposing the response at all angles from 0° to 360° and carrying out synchronous PSD analysis allows the modal direction to be located according to the spectrum energy distribution characteristics. When the rotation angle θ coincides with the normal coordinate axis, the PSD energy in the corresponding direction is concentrated at a single fundamental frequency. This enables the two orthogonal first-order modal directions of the structure to be accurately identified and modal decoupling to be realised.

**Figure 11:**
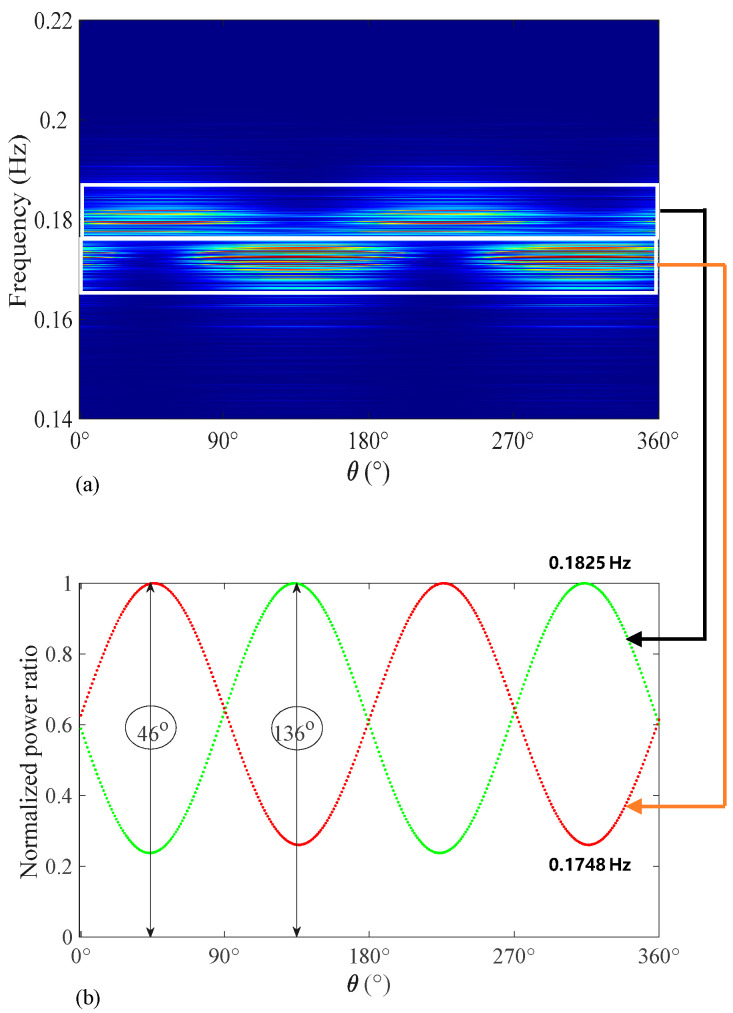
PSD of directional responses: (**a**) energy distribution with varying θ; (**b**) normalized energy proportion with varying θ.

As Figure 11 shows, the results of the normal mode decomposition clearly demonstrate the distribution characteristics of the two coupled first-order modes with respect to the rotation angle θ, achieving effective decoupling of the modes. The PSD spectrogram of the directional response shows that the energy of the two modes, which have similar frequencies of 0.1748 Hz and 0.1825 Hz, alternates and dominates as the θ angle changes, demonstrating periodic energy transfer characteristics. The normalised power ratio curve quantifies this process further: the energy of the 0.1825 Hz mode peaks at θ = 46°, while the energy of the 0.1748 Hz mode is concentrated at θ = 136°. The main energy directions of the two modes differ by 90°, which conforms to the orthogonality of the first-order sway modes of the structure.

When θ = 46° or 136°, the energy is entirely concentrated at the corresponding fundamental frequency, thus determining that the normal modal coordinate axis of the structure rotates counterclockwise by 46° relative to the observation coordinate axis. Following decoupling, both modes exhibit a single, frequency-dominated response in their respective normal directions, with highly concentrated spectrum energy. This effectively eliminates the modal aliasing effect under the observation coordinate system.

### 3.7. Modal Decoupling Verification Analysis

Once the deflection angle of the normal coordinate axis has been determined relative to the observation coordinate axis, these closely coupled modes can be separated by transforming the response into the normal coordinate axis.

**Figure 12:**
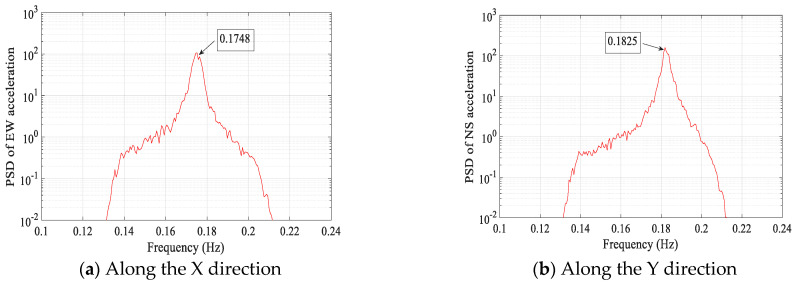
Elimination of beating in the frequency-domain: PSD of acceleration responses under normal coordinates.

**Figure 13:**
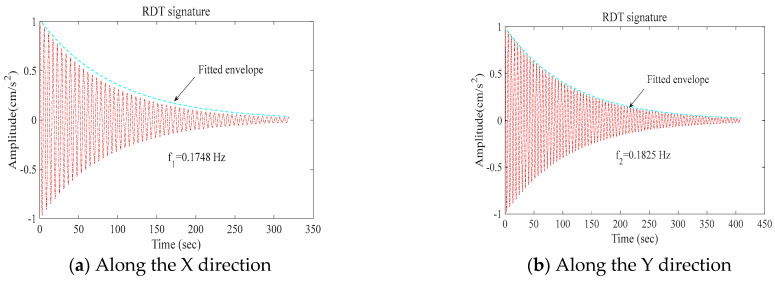
Elimination of beating in the time-domain: RDS of acceleration responses under normal coordinates.

Following normal mode decomposition, frequency- and time-domain analyses under the normal coordinate system clearly demonstrate the successful decoupling of the coupled modes. In the frequency-domain (Figure 12), the power spectral density (PSD) curves of the two directions show a single main energy peak, corresponding to frequencies of 0.1825 Hz and 0.1748 Hz, respectively. This eliminates the double-peak aliasing phenomenon observed in the observation coordinate system, with each curve dominating the response in its respective normal direction without interference from cross-frequency components. In the time-domain RDT analysis (Figure 13), the free decay signals of the two directions both exhibit typical single-mode decay characteristics: the amplitude decays smoothly along an exponential envelope and there is no periodic beat oscillation under the observation coordinates, which further verifies that the modal coupling effect has been effectively eliminated.

### 3.8. Structural Modal Parameter Identification

**Figure 14:**
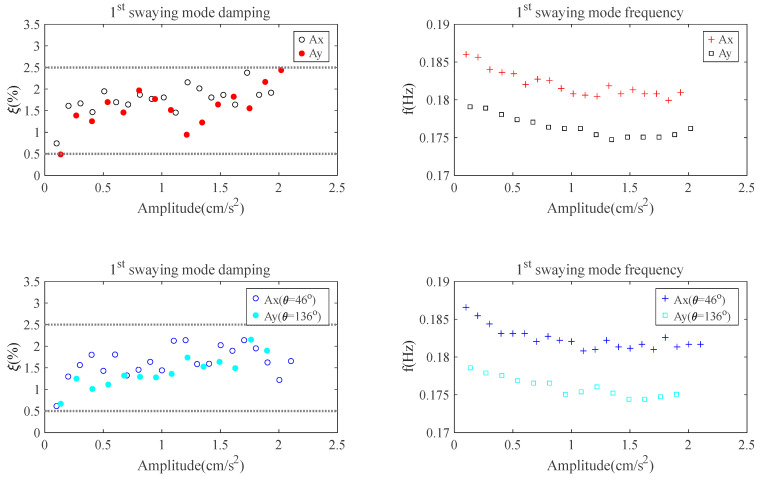
Comparative analysis of structural modal parameter identification (the dotted lines show the range of parameter variations).

As shown in Figure 14, the identified modal damping ratios and frequencies were obtained by applying RDT to the decoupled acceleration responses, after carrying out a normal modal decomposition (i.e., decoupling via coordinate rotation). This involved extracting the free decay signals, fitting the damping ratios using a logarithmic decay method and calculating the modal frequencies based on time-domain peak intervals. When the normal modal decomposition method is used to achieve dense modal decoupling, the first-order sway mode of the ZHCT exhibits a clear, amplitude-dependent evolution trend. Identification accuracy and stability are significantly better under the normal coordinate system than under the viewing coordinate system, effectively correcting the systematic parameter deviation caused by modal coupling.

Overall, the first-order sway modal frequency of the ZHCT is centered at 0.18 Hz, with a primary fluctuation range of 0.175 to 0.185 Hz. Its first-order sway damping ratio is centered at 1.5%, with a primary fluctuation range of 1 to 2%. These ranges of modal parameters are consistent with the results of the previous study [35], which reported the first-order sway frequencies in two directions obtained from field measurements under normal wind conditions as 0.182 Hz and 0.184 Hz, respectively. Furthermore, another relevant study [36] conducted a specific analysis of the amplitude dependence of the modal parameters based on field measurement data collected during Typhoon Cempaka (No. 2107). This study indicated that the first-order sway modal frequency fluctuated between 0.178 Hz and 0.186 Hz, while the damping ratio fluctuated between 0.5% and 1.5%. The findings of these existing studies on the ZHCT are highly consistent with the results of this study, thereby validating the reliability of the results obtained.

In terms of the damping ratio, the first-order modal damping ratio under both coordinate systems shows an overall upward trend as the vibration amplitude increases (from 0 to 2.5 cm/s^2^), with the variation range concentrated within 0.5% to 2.5%. This is highly consistent with the conclusions reported in the literature on similar super-high-rise buildings worldwide, indicating the basic rationality of the results [37,38]. However, there is a significant difference in discreteness between the two coordinate systems: the damping ratio data points under the observation coordinate system are more scattered and easily disturbed by the beat effect caused by modal coupling. This results in the periodic fluctuation of amplitude being misjudged as energy dissipation of the structure, leading to an overestimation of the damping ratio. In contrast, the damping ratio data points under the normal coordinate system are more concentrated, eliminating the false contribution of the beat effect. This produces identification results that are closer to the actual structural damping characteristics and are more closely aligned with the reasonable intervals reported in the literature.

In terms of natural frequency, the first-order modal frequency under both coordinate systems exhibits slight nonlinear amplitude dependence. When the vibration acceleration amplitude increases from 0 to approximately 2.5 cm/s^2^, the frequency decreases slowly by around 0.005 Hz overall, reflecting the softening of the stiffness of super high-rise buildings under strong winds [39,40]. While the frequency values under the two coordinate systems differ only slightly, the frequency data under the normal coordinate system converges more quickly and more accurately reflects the tendency of the structural natural frequency to evolve with vibration intensity.

Generally, the parameter identification results after modal decoupling reveal that the first-order modal damping ratio of super high-rise buildings increases with amplitude and that the frequency decreases with amplitude. They also clarify the adverse effect of the beat effect on modal parameter identification through coordinate system comparison.

## 4. Concluding Remarks

This study, based on field measurements of wind-induced responses obtained from the supertall ZHCT during Super Typhoon Saola, systematically examines the mechanisms underlying modal mixing and its impact on structural dynamics. The main conclusions are as follows:

1. The non-stationary and anisotropic characteristics of the wind-induced response in the tall building with irregular cross-sections are demonstrated based on field measurement data from the ZHCT under super typhoon excitations, with response peaks showing a high degree of synchronisation with the evolution of the typhoon’s intensity.
2. Cross-validation of frequency-domain PSD and time-domain RDT confirmed significant modal mixing and beat effects in the viewing coordinate system. This is fundamentally caused by a 46° angle of deviation between the viewing coordinate system and the normal coordinate system. The coupling between two adjacent modes (0.1748 Hz and 0.1825 Hz) results in a bimodal characteristic in the frequency-domain and a beat frequency period of approximately 260 s in the time-domain. Additionally, theoretical calculations closely align with the measured results.
3. Employing the normal modal decomposition method and applying a coordinate rotation transformation effectively decoupled the close modes, separating the coupled dual-modal response into two orthogonal single-modal responses. Following decoupling, dispersion in the identification of damping ratios and frequencies was significantly reduced. The results revealed an amplitude-dependent evolution pattern, whereby the damping ratio increases and the frequency decreases with increasing amplitude.
4. The limitations of the decoupling strategy employed in this study include: reliance on the simultaneous presence of two closely spaced modes; for structures dominated by a single mode or with a large separation in modal frequencies, the aliasing effect may not be significant; estimation of beat frequency periods requires a long period of steady-state response, which may be unstable during the initial or decay phases of non-steady typhoons. Furthermore, future research should focus on identifying three-dimensional (torsional) modes in different directions, tracking time-varying modal directions and developing adaptive decoupling algorithms that do not require manual judgement.

## Figures and Tables

**Table 1:** Information on sensors deployed on the ZHCT.

Sensor	Location	Type	No.	Range	Accuracy
Accelerometer	56F	AS-301C2W6	2 per floor	±2 g	±0.005%
Velocimeter	56F	991B	2 per floor	0.7 m/s	2 × 10^–6^
Pressure transducer	56F	Setra268	10	±2.5 kPa	±0.07% FS

**Table 2:** Beating periods identified from PSD and RDT.

Direction	PSD			RDT	Difference (%)
	f_1_ (Hz)	f_2_ (Hz)	T_b_ PSD (s)	T_b_ RDT (s)	
X	0.1748	0.1825	260	265	1.92
Y	0.1825	0.1748	260	260	0

Note: Difference = (Tb|RDT − Tb|PSD)/Tb|PSD.

## Data Availability

Data will be made available on request.

## Abbreviations

The following abbreviations are used in this manuscript:

ZHCT	Zhuhai Center Tower
RDT	Random Decrement Technique
PSD	Power Spectral Density
SHM	Structural Health Monitoring
BSDA	Bayesian Spectral Density Approach
CTBUH	Council on Tall Buildings and Urban Habitat
EMA	Experimental Modal Analysis
ERA	Eigensystem Realization Algorithm
HHT	Hilbert–Huang Transform
LiDAR	Light Detection and Ranging
NExT	Natural Excitation Technique
OMA	Operational Modal Analysis
PP	Peak Picking
SSI-COV	Covariance-Driven Stochastic Subspace Identification
